# Application of polyetheretherketone temporal muscle prostheses for temporal hollowing following cranioplasty

**DOI:** 10.3389/fneur.2025.1664624

**Published:** 2025-09-16

**Authors:** Xiaoyu Yang, Yi Zhang, Junwen Guan

**Affiliations:** ^1^Department of Neurosurgery, West China Hospital, Sichuan University, Chengdu, China; ^2^Department of Neurosurgery, Chengdu Women's and Children's Central Hospital, School of Medicine, University of Electronic Science and Technology of China, Chengdu, China

**Keywords:** polyetheretherketone, temporal hollowing, cranioplasty, prostheses, 3D-printed

## Abstract

**Background:**

Decompressive craniectomy is an effective intervention for refractory intracranial hypertension. However, subsequent cranioplasty, though technically less demanding, is associated with a non-negligible incidence of complications. Among these, temporal hollowing is a common yet frequently overlooked sequela that significantly compromises facial aesthetics and can lead to various psychosocial issues.

**Methods:**

Based on the pathophysiological mechanisms underlying temporal hollowing following cranioplasty and incorporating clinical experience from West China Hospital, Sichuan University, this study explores the application of 3D-printed polyetheretherketone (PEEK) temporal muscle prostheses for correcting temporal hollowing after 3D-printed PEEK cranioplasty. The approach involves precise measurement of the contralateral temporal muscle dimensions and volumetric assessment of the atrophied ipsilateral muscle to fabricate patient-specific 3D-printed PEEK implants. These customized prostheses are then surgically implanted in a secondary procedure to restore temporal contour symmetry.

**Conclusion:**

Temporal hollowing represents a frequent sequela of cranioplasty, arising from multifactorial mechanisms and often contributing to psychosocial challenges. The patient-specific 3D-printed PEEK temporal muscle prosthesis, developed based on contralateral temporal muscle metrics at West China Hospital, offers a precise and individualized solution for restoring temporal contour symmetry.

## 1 Introduction

Decompressive craniectomy is an effective surgical intervention for refractory intracranial hypertension, while the subsequent secondary cranioplasty, although technically less demanding, carries a non-negligible complication rate, among which temporal hollowing represents a common yet frequently overlooked sequela. It can significantly impair facial aesthetics, leading to various psychosocial issues (1). Therefore, based on the pathomechanisms of temporal hollowing and incorporating clinical experience from West China Hospital, Sichuan University, this article discusses the application of 3D-printed polyetheretherketone (PEEK) temporal muscle prostheses for addressing temporal hollowing following 3D-printed PEEK cranioplasty.

## 2 Etiology and mechanisms of temporal hollowing

Temporal hollowing is primarily caused by temporal muscle atrophy. Temporal muscle atrophy refers to the loss of volume, thickness, or function of the temporal muscle, commonly observed after decompressive craniectomy and subsequent cranioplasty. Its pathogenesis primarily involves direct surgical trauma, neurogenic atrophy due to denervation, adhesion formation and fibrosis, diminished blood supply and local ischemia, systemic inflammation and infection, as well as disuse atrophy. Specifically, intraoperative dissection often necessitates stripping, stretching, or incision of the temporal muscle to expose the surgical field. This direct trauma can cause muscle fiber damage and subsequent atrophy; for instance, intraoperative manipulation (e.g., stretching or incision) may lead to temporomandibular joint disorders and chronic pain—findings linked to muscle injury (2). Handling the temporal muscle is a surgically challenging step, wherein excessive stretching or cutting during dissection can induce atrophy (3), while improper dissection risks devascularization or laceration, underscoring the imperative to “maximize protection of the temporal muscle” to mitigate damage (4). Concurrently, disruption or compression of trigeminal nerve branches innervating the temporal muscle can impair neural signaling, triggering neurogenic atrophy. In trigeminal neuralgia (TN) patients, neurovascular conflict (NVC) correlates significantly with temporal muscle atrophy (5). Furthermore, NVC serves as an imaging biomarker predicting temporal muscle atrophy, and intraoperative nerve stretching or compression disrupts neural signals—functional loss of the trigeminal nerve renders the muscle “nonfunctional,” resulting in dynamic atrophy. Although derived from facial palsy studies, this mechanism extends to potential nerve injury in decompressive craniectomy procedures (6). Additionally, adhesions frequently develop between the temporal muscle and dura or implants, immobilizing the muscle, restricting motion, and promoting fibrosis and atrophy—likely a consequence of local inflammatory responses. Adhesion formation is a common complication (particularly among the temporal muscle, dura, and brain surface), leading to muscle fixation and disuse atrophy (7). Discussions on using titanium mesh to reduce adhesions note that adhesions “compromise the integrity of the temporal muscle,” impeding contraction and nutrient supply (8). Diminished blood supply arises when intraoperative vascular injury (e.g., to the superficial temporal artery) causes ischemia, edema, or swelling, culminating in myocyte death and volume reduction (9). Systemic inflammation or postoperative infections (e.g., pneumonia or bloodstream infections) catalyze muscle catabolism, accelerating atrophy; in critically ill patients, infections correlate with more pronounced temporal muscle volume loss, as inflammatory cytokines promote protein degradation and muscle wasting (10). Temporal muscle atrophy observed post-cardiac arrest—though not directly associated with mortality—suggests inflammatory states (e.g., systemic ischemia–reperfusion injury) may constitute an independent mechanism (11). Muscle biopsies revealing “inflammatory cell infiltration and fibrosis” further corroborate this inflammation-mediated destructive process (12). Lastly, reduced postoperative masticatory activity or muscle immobilization serves as a secondary atrophic mechanism (13). Collectively, temporal hollowing results from the synergistic interplay of multiple pathological pathways.

## 3 Treatment of temporal hollowing

The treatment of temporal hollowing primarily falls into two main categories: surgical methods and injectable filler methods. Surgical methods involve reconstructing the temporal region structure, aiming to restore volume and contour, applicable to depressions caused by trauma, surgery (such as cranioplasty or craniotomy), or other factors; these methods reduce the hollowing by correcting muscle or soft tissue defects and include temporal muscle resuspension—a technique frequently used in cranioplasty that prevents depression caused by muscle retraction by re-securing the temporal muscle, for instance, novel resuspension techniques during cranioplasty can significantly minimize the incidence of temporal hollowing and reduce complication risks (13), with enhanced resuspension based on temporal muscle morphology effectively reducing postoperative depression during implementation; as well as the use of implants (including synthetic materials and autologous tissues), such as synthetic material implants (e.g., hydroxyapatite, polymethyl methacrylate (PMMA), or polyethylene) providing stable support and improving the depressed contour (14), or in cranial reconstruction, autologous bone grafts or adipo-dermal grafts (multilayered fat-dermis grafts) harvested from the suprapubic region and implanted into the temporal region effectively restore volume and contour, with this method exhibiting low complication rates and high patient satisfaction, although long-term outcomes require attention (15); furthermore, the use of soft-tissue volume augmentation implants created using 3D printing technology (3D-printed patient-specific implants) combined with virtual surgical planning enables precise reconstruction, restoring the defect immediately postoperatively with long-term follow-up demonstrating good functional and aesthetic outcomes (16, 17) for large-area defects, autologous flap transfers such as latissimus dorsi muscle free flaps can be used, repairing temporal defects via microsurgical anastomosis, a method particularly suitable for hollowing resulting from trauma or tumor resection, providing volume augmentation and long-term stability (18). Injectable filler methods fill the depression in a minimally invasive manner, typically used for mild to moderate hollowing or as surgical adjuncts, emphasizing anatomical knowledge and safe injection planes; these methods improve aesthetic appearance through volume augmentation, exhibit low complication rates, and good patient satisfaction, including autologous fat grafting (AFG)—a commonly used and effective method involving harvesting fat from the patient (e.g., abdomen) and transplanting it to the temporal region, where Doppler ultrasound (DUS)-guided AFG is particularly recommended as it allows precise targeting of temporal fat compartments (such as the superficial temporal fat pad), enabling large-volume augmentation and improving safety and efficacy, with AFG reducing complication rates and yielding high patient satisfaction; as well as the use of hyaluronic acid (HA) fillers such as VYC-20 L (used for improving temple hollowing), proven through randomized controlled trials to significantly reduce hollowing with good objective efficacy assessment and high subjective patient satisfaction (19), amino acid cross-linked hyaluronic acid (ACHA) demonstrated safe and effective for treating temporal hollowing in clinical studies, suitable for moderate to severe cases (20), HA-V and other hyaluronic acid products like the HA-V filler used for bilateral severe hollowing, proven effective in prospective studies with persistent volume improvement observed at 16-week follow-up (13) [requiring careful attention to anatomical structures such as the superficial temporal artery and facial nerve branches during injection to avoid complications (21)]; besides these, injectable methods also include other fillers such as stromal vascular fraction (SVF) gel (containing adipose-derived stem cells and extracellular matrix) used for age-related hollowing, SVF gel injections improve volume loss and are suitable for Asian populations, though long-term efficacy requires further study (16); injection techniques emphasize the importance of an anatomical approach: the injection plane can be chosen as subfascial, within the superficial temporal fat pad, or submuscular to avoid the superficial temporal artery, facial nerve branches, and sentinel vein, ensuring safety (22). Overall, treatments for temporal hollowing are diverse; surgical methods are suitable for structural defects or severe hollowing, providing long-lasting, stable results, while injectable methods manage volume loss minimally invasively, safely and effectively; the treatment choice should be based on the specific etiology and patient needs, underscoring the importance of anatomical knowledge to mitigate risks.

## 4 Clinical experience from West China Hospital, Sichuan University

**Figure 1:**
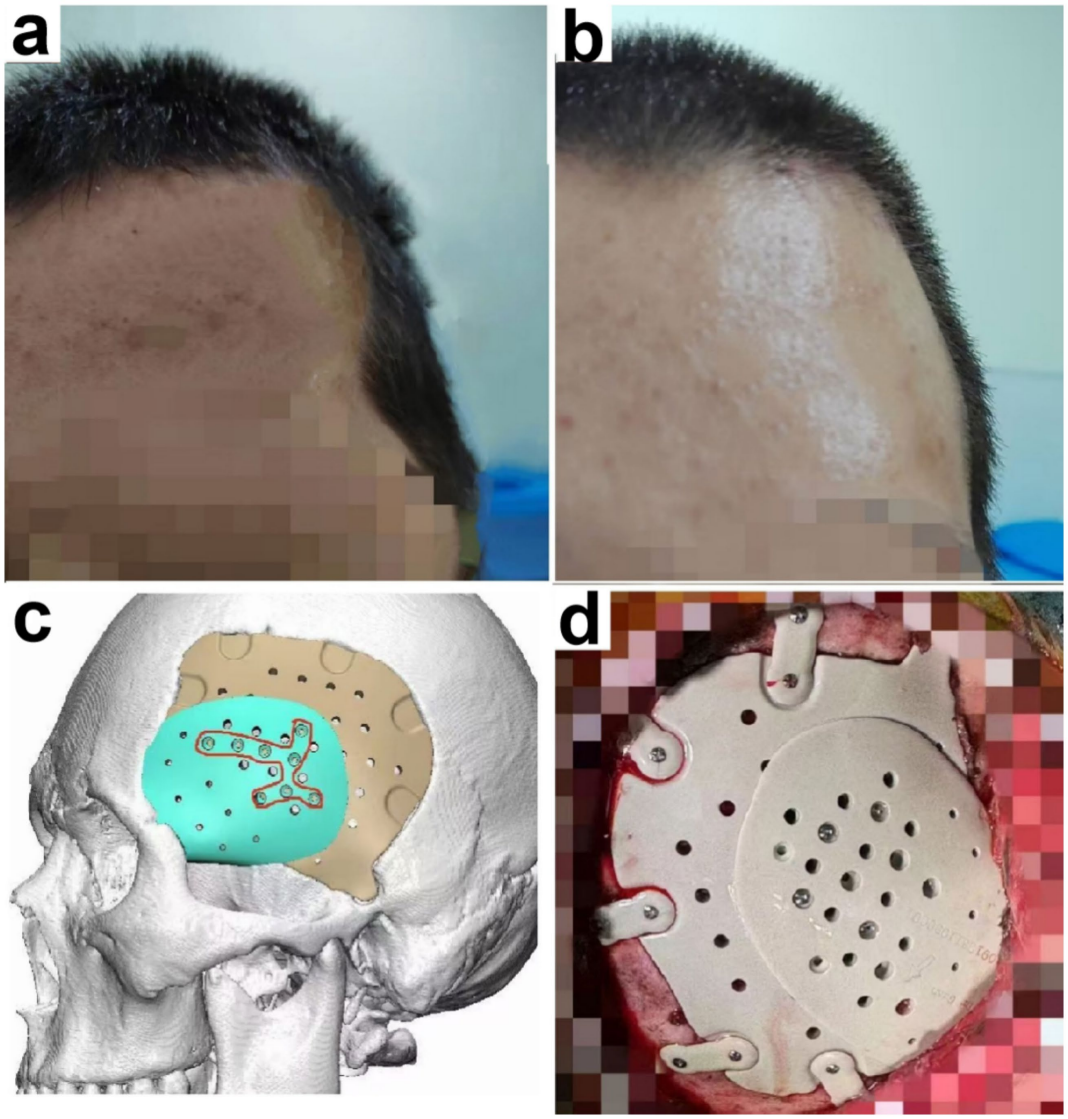
Application of polyetheretherketone temporal muscle prostheses for temporal hollowing following cranioplasty. **(a)** Pre-operative photos. **(b)** Post-operative photos. **(c)** PEEK temporal muscle prosthesis design diagram. **(d)** Intra-operative photos.

PEEK has become one of the most commonly used artificial materials for cranioplasty in China due to its high histocompatibility, favorable physical properties, and high malleability. However, many surgeons fail to consider temporal muscle atrophy when designing PEEK implants, leading to postoperative dissatisfaction with appearance among some patients. For such patients, revision surgery can be performed to fill the temporal hollowing (Figure 1). First, we obtain the patient’s imaging data to accurately measure the volume and shape of the bilateral temporal muscles, thereby determining the volume and shape of the missing temporal muscle on the affected side. Using this data, a 3D-printed PEEK temporal muscle prosthesis is fabricated and surgically fixed onto the surface of the previously implanted PEEK cranial implant. Postoperatively, the temporal region appears full, and patients are satisfied with their appearance. For a recent patient who underwent the procedure, a 6-month postoperative follow-up was conducted, revealing a full temporal contour without recurrence of hollowing and no other surgical complications. We summarize the experience with PEEK cranioplasty of West China Hospital as follows: 1. Temporal muscle atrophy must be considered during the design of PEEK cranial implants, and appropriate adjustments should be made; 2. For patients who have developed temporal hollowing after PEEK cranioplasty, revision surgery with a 3D-printed PEEK temporal muscle prosthesis can be performed.

## 5 Discussion and conclusion

Temporal hollowing, a prevalent sequela after cranioplasty (1), stems from multifactorial mechanisms including surgical trauma, neurogenic atrophy, ischemia, and systemic inflammation (2). While current treatments range from surgical interventions (e.g., muscle resuspension, autologous grafts, and 3D-printed implants) to minimally invasive fillers (e.g., ultrasound-guided fat grafting and tailored HA products) (13), challenges persist in achieving durable volume restoration with low morbidity. The clinical experience from West China Hospital introduces a precision-based solution: patient-specific 3D-printed PEEK prostheses fabricated using contralateral temporal muscle metrics. This approach addresses symmetry loss through individualized tissue-level restoration, leveraging PEEK’s biocompatibility and structural stability to mitigate traditional implant limitations. Crucially, optimizing outcomes demands neurovascular anatomy mastery to avoid injury during filler injections or surgical dissection (22). Future efforts should refine preventative strategies during initial craniectomy (e.g., nerve-sparing techniques) and validate long-term efficacy of PEEK prostheses. Integrating etiology-specific protocols with emerging biomaterials offers the most promising pathway for functional and aesthetic recovery.

## Data Availability

The original contributions presented in the study are included in the article/supplementary material, further inquiries can be directed to the corresponding author.
